# Quantitative MRI Biomarkers of Stereotactic Radiotherapy Outcome in Brain Metastasis

**DOI:** 10.1038/s41598-019-56185-5

**Published:** 2019-12-27

**Authors:** Elham Karami, Hany Soliman, Mark Ruschin, Arjun Sahgal, Sten Myrehaug, Chia-Lin Tseng, Gregory J. Czarnota, Pejman Jabehdar-Maralani, Brige Chugh, Angus Lau, Greg J. Stanisz, Ali Sadeghi-Naini

**Affiliations:** 1 Department of Electrical Engineering and Computer Science, Lassonde School of Engineering, York University, Toronto, ON Canada; 2 Department of Medical Biophysics, University of Toronto, Toronto, ON Canada; 3 Physical Sciences Platform, Sunnybrook Research Institute, Sunnybrook Health Sciences Centre, Toronto, ON Canada; 4 Department of Radiation Oncology, Odette Cancer Centre, Sunnybrook Health Sciences Centre, Toronto, ON Canada; 5 Department of Radiation Oncology, University of Toronto, Toronto, ON Canada; 6 Department of Medical Imaging, Sunnybrook Health Sciences Centre, Toronto, ON Canada; 7 Department of Neurosurgery and Pediatric Neurosurgery, Medical University, Lublin, Poland

**Keywords:** CNS cancer, Predictive markers, Prognostic markers, Biomedical engineering, Computational science

## Abstract

About 20–40% of cancer patients develop brain metastases, causing significant morbidity and mortality. Stereotactic radiation treatment is an established option that delivers high dose radiation to the target while sparing the surrounding normal tissue. However, up to 20% of metastatic brain tumours progress despite stereotactic treatment, and it can take months before it is evident on follow-up imaging. An early predictor of radiation therapy outcome in terms of tumour local failure (LF) is crucial, and can facilitate treatment adjustments or allow for early salvage treatment. In this study, an MR-based radiomics framework was proposed to derive and investigate quantitative MRI (qMRI) biomarkers for the outcome of LF in brain metastasis patients treated with hypo-fractionated stereotactic radiation therapy (SRT). The qMRI biomarkers were constructed through a multi-step feature extraction/reduction/selection framework using the conventional MR imaging data acquired from 100 patients (133 lesions), and were applied in conjunction with machine learning techniques for outcome prediction and risk assessment. The results indicated that the majority of the features in the optimal qMRI biomarkers characterize the heterogeneity in the surrounding regions of tumour including edema and tumour/lesion margins. The optimal qMRI biomarker consisted of five features that predict the outcome of LF with an area under the curve (AUC) of 0.79, and a cross-validated sensitivity and specificity of 81% and 79%, respectively. The Kaplan-Meier analyses showed a statistically significant difference in local control (p-value < 0.0001) and overall survival (p = 0.01). Findings from this study are a step towards using qMRI for early prediction of local failure in brain metastasis patients treated with SRT. This may facilitate early adjustments in treatment, such as surgical resection or salvage radiation, that can potentially improve treatment outcomes. Investigations on larger cohorts of patients are, however, required for further validation of the technique.

## Introduction

Brain metastasis is the most common intracranial malignancy in both men and woman^1^. Despite advances in surgical, systemic and radiotherapy treatments, patients with brain metastasis still have poor overall survival (OS) and quality of life^2,3^. Treatment options for brain metastases include whole brain radiation therapy (WBRT), single fraction stereotactic radiosurgery (SRS), hypo-fractionated stereotactic radiation therapy (SRT), surgical resection, and systemic therapy^4,5^. The traditional treatment paradigm for patients with brain metastasis was WBRT. However, WBRT has increasingly been replaced with focused radiation techniques such as SRS or SRT to spare the cognitive and quality of life impacts of WBRT in patients with a limited number of brain metastases^6–11^. Nonetheless, between 10–20% of tumours progress locally after stereotactic radiation treatment^12–16^. Local response to stereotactic radiation treatment is conventionally evaluated based on changes in tumour size using anatomical/structural magnetic resonance imaging (MRI). However, changes in physical dimensions of a treated tumour may take months before it is evident on follow-up images. Furthermore, early changes in size are not always correlated with the long-term local control (LC) of the tumour. Predicting LF of tumours early after SRT can facilitate effective treatment adjustments and potentially lead to improved treatment outcomes.

The precision medicine paradigm in healthcare decision-making has shown promise to improve cancer treatment outcomes^17–19^. Biomarkers from genomic and proteomic data derived in primary cancers of the lung, breast, ovary or brain can be used to stratify patients into different diagnostic/prognostic groups leading to more-effective treatment paths^20–25^. Acquiring genomic and proteomic biomarkers are, however, challenging in terms of implementation as they are invasive, not always technically feasible, and in some cases not robust as they may not represent the entirety of the tumour^26,27^. A reason for this observation is the fact that tumours are often spatially heterogeneous, and hard to characterize completely using core biopsy specimens^28,29^.

A number of recent studies have proposed quantitative imaging techniques to characterize different malignancies, including breast, lung, prostate and brain cancer, and monitor/predict their response to anti-cancer therapies^30–39^. To identify the quantitative imaging biomarkers with high sensitivity and specificity in such applications, computational methods have been adapted through an emerging field of research named radiomics^40–44^. The radiomic analysis often utilizes the images obtained as part of the standard of patient care, and can be implemented in the clinical routines with minimal overload. Further, the 3D volumetric images frequently applied in radiomics permits a comprehensive assessment of the entire tumour volume and surrounding regions. Recent studies have reported strong links between the radiomic signature of various tumours and their phenotype and genomic and proteomic profiles^45–47^. Aert *et al*. demonstrate that the computed tomography (CT) based radiomic features characterizing the spatial heterogeneity in lung and head and neck tumours are associated with underlying gene-expression patterns^45^. In another study, Zhu *et al*. indicate that in invasive breast cancer the transcriptional activities of various genetic pathways and miRNA expressions are associated with the tumour shape and texture in dynamic contrast-enhanced magnetic resonance (MR) images^46^. Similarly, Grossmann *et al*. report that the intra-tumour heterogeneity and intensity dispersion in CT images acquired from lung cancer patients are associated with the molecular characteristics of the tumour, including the activity of RNA polymerase transcription and the autodegration pathway of a ubiquitin ligase^47^.

A number of previous studies have investigated the efficacy of different radiomic biomarkers in cancer diagnosis, treatment response monitoring, and outcome prediction^48–53^. A study by Gómez Flores *et al*. suggests that ultrasound radiomic features can be used to characterize benign versus malignant breast lesions^48^. Li *et al*. demonstrate the potential of MR radiomic features to predict OS in Glioblastoma multiform^49^. Other studies have correlated radiomic biomarkers derived from positron emission tomography (PET) and CT images with loco-regional recurrence (LR), distance metastasis (DM), and OS in head and neck^50^, and lung cancer patients^51^.

**Figure 1:**
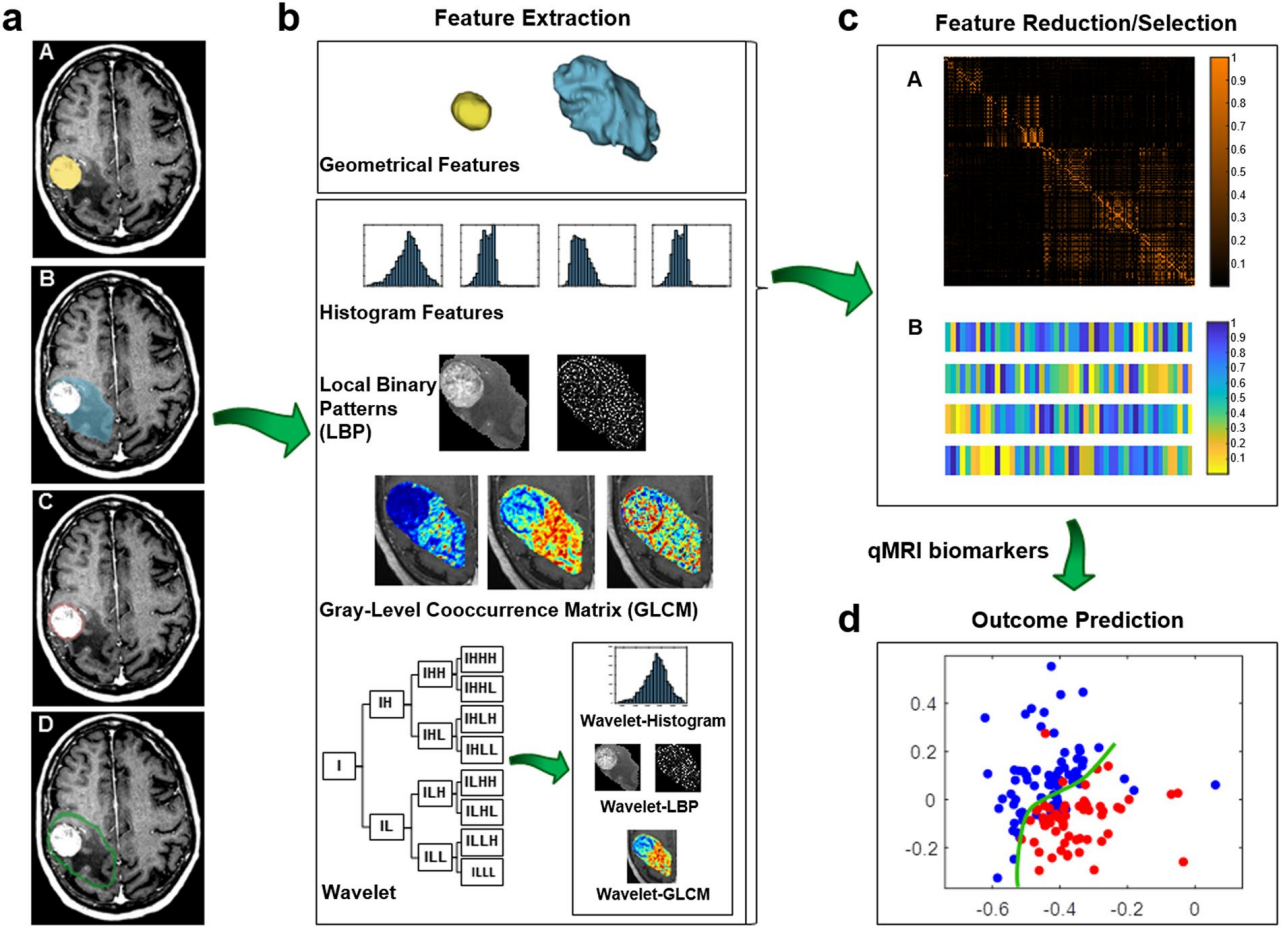
Scheme of the radiomics-based outcome prediction framework. (**a**) The binary masks including the tumour delineated by expert oncologists (A), edema segmented semi-automatically (B), tumour-margin (C), and the lesion-margin (D). (**b**) Extracting the geometrical and textural features from T1w and T2-FLAIR images within the binary masks. (**c**) Correlation-based feature reduction (A), and multi-step feature selection (B). (**d**) Outcome prediction (LC versus LF) using the SVM classifier.

In this study, quantitative MRI (qMRI) biomarkers was investigated through a radiomics analysis framework (Fig. 1) to predict the outcome of LF in brain metastasis patients treated with SRT. MR images were acquired from 100 patients (133 tumours) triaged for hypo-fractionated SRT before and within three months after the treatment, as part of our institutional standard of care. Various geometrical and textural features were extracted from gadolinium-contrast-enhanced-T1-weighted (T1w) and T2-weighted-fluid-attenuation-inversion-recovery (T2-FLAIR) images within the tumour and edema regions and the corresponding margins. A multi-step feature reduction and selection method consisting of correlation-based feature reduction, feature ranking, and forward feature selection based on bootstrap 0.632 + area under the curve (AUC) was used to construct the optimal qMRI biomarkers (consisted of four or five features). The feature selection results demonstrate that the qMRI biomarkers extracted from the edema, tumour margin and the lesion margin have more prognostic power compared to features extracted from the tumour itself. A support vector machine (SVM) classifier was used to predict the SRT outcome in terms of LC or LF. The results demonstrate a good potential of qMRI biomarkers to predict the survival-linked LC/LF in brain metastasis patients treated with SRT.

## Results

Figure 1 demonstrates the scheme of the radiomics analysis framework applied in this study to develop qMRI signatures of metastatic brain lesions treated with SRT for the LC outcome evaluation. A total of 3072 imaging features were extracted from four sub-regions of each lesion: the tumour, edema, tumour-margin, and the lesion-margin, as described in the Methods.

**Figure 2:**
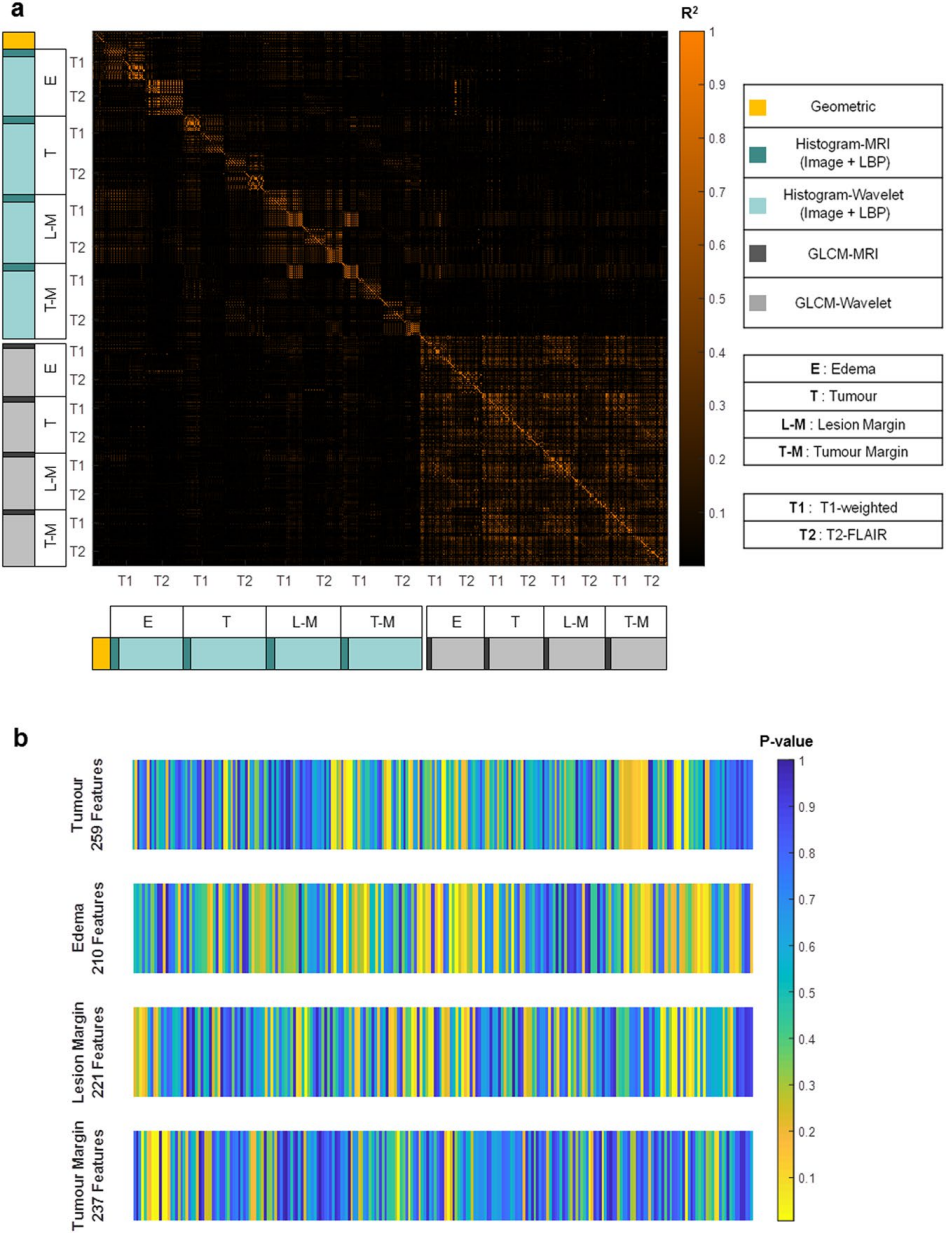
The qMRI feature heat maps. (**a**) The R-squared heat map generated using the Pearson correlation coefficients for all pairs of the extracted features. (**b**) The p-value heat maps for different sub-regions of the lesion generated using the Mann-Whitney U test (overall LC versus LF) for each feature after the redundant features were eliminated.

The heat maps of the derived qMRI features are detailed in Fig. 2. Figure 2a shows the R-squared heat map generated using the Pearson correlation coefficient obtained for each pair of the extracted features. The heat map identifies several clusters of redundant features with high levels of inter-feature correlation. An R-squared threshold of 0.8 was used to reduce clusters of highly-correlated features to a single representative feature with the largest natural dynamic range. This process reduced the number of features from 3072 to 927. A non-parametric Mann-Whitney U test was applied to quantify the level of statistical difference exhibited by each feature between the lesions with different local outcomes (LC versus LF). Figure 2b demonstrates the p-value heat maps obtained for different sub-regions of the lesion after the redundant features are eliminated. The heat maps highlight several qMRI features demonstrating a statistically significant difference between the lesions with an overall LC versus LF outcome (p-value < 0.05). Similar results were obtained for the 6-month and 12-month LC/LF outcomes (Supplementary Figures 1 and 2).

The Mann-Whitney U test was used in conjunction with a 50-fold sampling scheme to rank the features for the overall, 6-month, and 12-month LC/LF outcomes, followed by a forward feature selection with bootstrap 0.632 + AUC $({\widehat{AUC}}_{.632+})$. Table 1 demonstrates the optimal qMRI biomarkers obtained for each outcome. The optimal qMRI biomarker consists of five features for the overall LC/LF outcome, and four features for the 6-month and 12-month outcomes. Among the 13 distinct features selected, four features (LM-W_LHL_-G-MaxProb-T2, TM-W_LHL_-G-MaxProb-T2, LM-W_HLL_- G-MaxProb-T2, E-W_HLH_-H-Max-T2) were statistically extremely significant (p-value < 0.001), seven features (E-W_HHL_-H-Max_T2, T-H-Min-T2, TM-W_HLH_-H-Min-T1, E-MedianLBP-T1, T-MedianLBP-T1, E-W_HLH_-H-MeanAbsDev-T2, TM-GE-Convexity-T1) were statistically highly significant (p-value < 0.01), and two features (TM-W_HLH_-H-Min-T2, LM-W_HLL_-H-Range-T2) were statistically significant (p-value < 0.05), between the LC versus LF outcomes. The selected features mainly characterize the heterogeneity in the surrounding regions of tumour including edema, tumour-margin, and lesion-margin.

**Table 1:** The optimal qMRI biomarkers obtained for each outcome.

Outcome	Selected Features	p-value
Overall LC/LF	Lesion-Margin_Wavelet_I_LHL__GLCM_MaximumProbability_T2 **(LM-W**_**LHL**_**-G-MaxProb-T2)**	0.0003
	Edema_Wavelet_I_HHL__Histogram_Maximum_T2 **(E-W**_**HHL**_**-H-Max_T2)**	0.002
	Tumour_Histogram_Minimum_T2 **(T-H-Min-T2)**	0.007
	Tumour-Margin _Wavelet_I_HLH__Histogram_Minimum_T2 **(TM-W**_**HLH**_**-H-Min-T2)**	0.02
	Lesion-Margin _Wavelet_I_HLL__Histogram_Range_T2 **(LM-W**_**HLL**_**-H-Range-T2)**	0.03
6-Month LC/LF	Tumour-Margin _Wavelet_I_LHL__GLCM_MaximumProbability_T2 **(TM-W**_**LHL**_**-G-MaxProb-T2)**	0.0001
	Tumour-Margin _Wavelet_I_HLH__Histogram_Minimum_T1 **(TM-W**_**HLH**_**-H-Min-T1)**	0.004
	Edema_LBP_Median_T1 **(E-MedianLBP-T1)**	0.002
	Tumour_LBP_Median_T1 **(T-MedianLBP-T1)**	0.002
12-Month LC/LF	Lesion-Margin_Wavelet_I_HLL__GLCM_MaximumProbability_T2 **(LM-W**_**HLL**_**- G-MaxProb-T2)**	0.00004
	Edema_Wavelet_I_HLH__Histogram_Maximum_T2 **(E-W**_**HLH**_**-H-Max-T2)**	0.00005
	Edema_Wavelet_I_HLH__Histogram_MeanAbsoluteDeviation_T2 **(E-W**_**HLH**_**-H-MeanAbsDev-T2)**	0.003
	Tumour-Margin _Geometric_Convexity_T1 **(TM-GE-Convexity-T1)**	0.007

**Figure 3:**
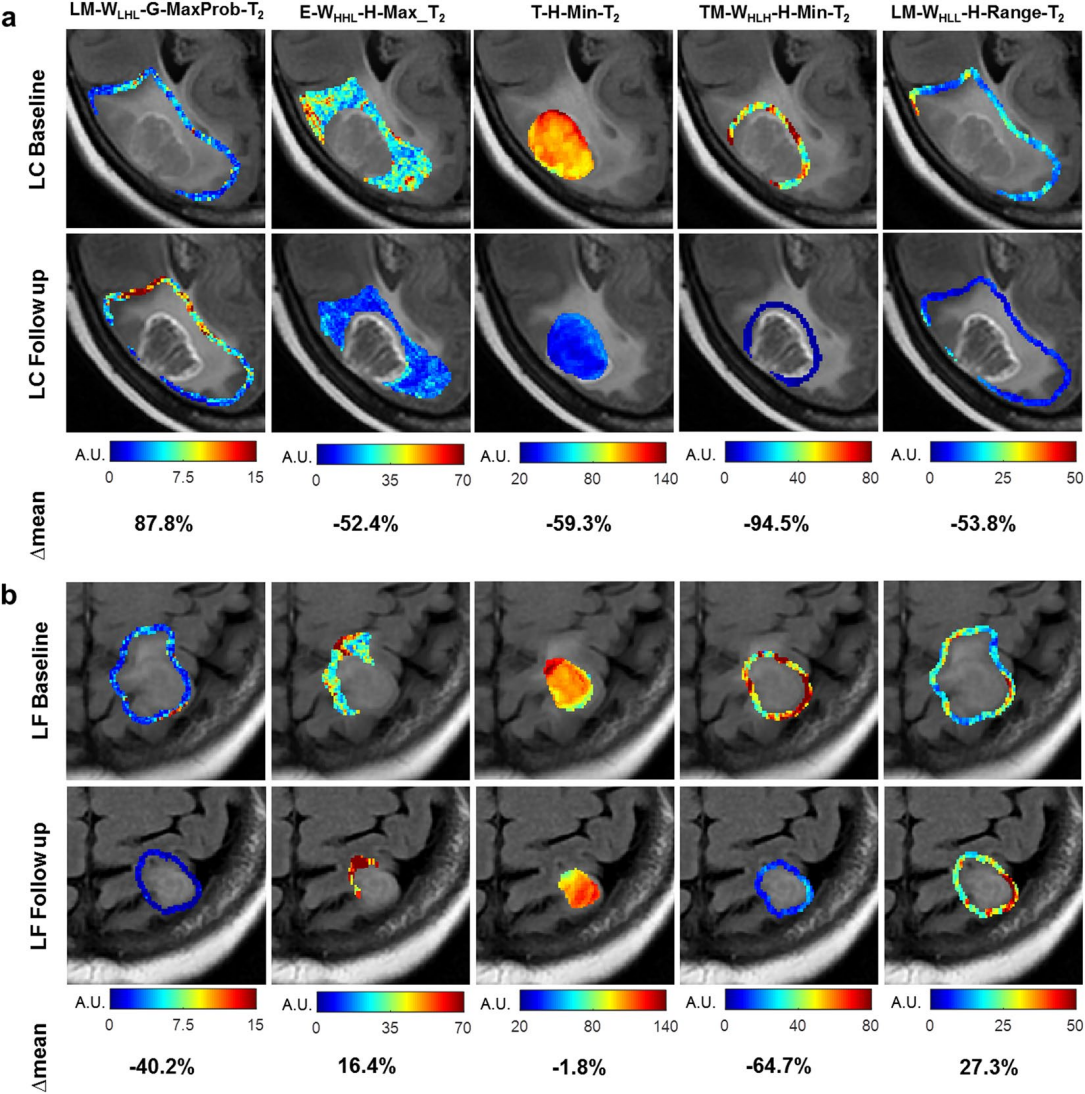
Representative parametric maps of the features in the optimal qMRI biomarker for the overall LC/LF outcome. The parametric maps show the spatial variations in the features derived from the MR images acquired at the baseline and the first follow up for representative tumours with LC (**a**) and LF (**b**) outcomes. The mean relative change from the baseline at the first follow up (∆mean) is given for each feature. Biomarker abbreviations: LM-W_LHL_-G-MaxProb-T2: Lesion-Margin_Wavelet_I_LHL__GLCM_MaximumProbability_T2; E-W_HHL_-H-Max_T2: Edema_Wavelet_I_HHL__Histogram_Maximum_T2; T-H-Min-T2: Tumour_Histogram_Minimum_T2; TM-W_HLH_-H-Min-T2: Tumour-Margin _Wavelet_I_HLH__Histogram_Minimum_T2; LM-W_HLL_-H-Range-T2: Lesion-Margin _Wavelet_I_HLL__Histogram_Range_T2.

Figure 3 contrasts two representative tumours, one with LC on long-term follow up (Fig. 3a) and the other one with a LF (Fig. 3b). The parametric maps at first follow up show significantly different changes from the baseline between the tumours. Specifically, the LM-W_LHL_-G-MaxProb-T2, E-W_HHL_-H-Max_T2, T-H-Min-T2, TM-W_HLH_-H-Min-T2, and LM-W_HLL_-H-Range-T2 parametric maps demonstrate a mean change of 88% versus −40%, −52% versus 16%, −59% versus −2%, −95% versus −65%, and −54% versus 27%, respectively, for the LC vs LF case, respectively.

### Early prediction of SRT outcome

Table 2 summarizes the results of LC/LF outcome prediction using the optimal qMRI biomarkers with an SVM classifier. The table presents the prediction results in terms of ${\widehat{AUC}}_{.632+}$ as well as the leave-one-patient-out (LOPO) cross-validated sensitivity, specificity, and accuracy. The maximum ${\widehat{AUC}}_{.632+}$ was 0.82, using 7 features for the 6-month and overall LC/LF, and 6 features for the 12-month local outcome. The optimal qMRI biomarkers (the smallest set of features as presented in Table 1) resulted in an ${\widehat{AUC}}_{.632+}$ of 0.80, 0.81, and 0.79 for the 6-month, 12-month and overall LC/LF, respectively. In addition, the optimal qMRI biomarkers could predict the 6-month, 12-month and overall LC/LF outcomes with a cross-validated accuracy of 80%, 82% and 80%, respectively.

**Table 2:** The prediction results for overall, 6-month, and 12-month LC/LF outcomes.

Outcome	${\widehat{{\boldsymbol{AUC}}}}_{{\bf{.632}}{\boldsymbol{+}}}$ (Maximum)	${\widehat{{\boldsymbol{AUC}}}}_{{\bf{.632}}{\boldsymbol{+}}}$ (Optimal qMRI Biomarker)	Accuracy	Sensitivity	Specificity
Overall LC/LF	0.82 7 features	0.79 5 features	80%	81%	79%
6-Month LC/LF	0.82 7 features	0.80 4 features	80%	83%	79%
12-Month LC/LF	0.82 6 features	0.81 4 features	82%	80%	85%

### Risk assessment

**Figure 4:**
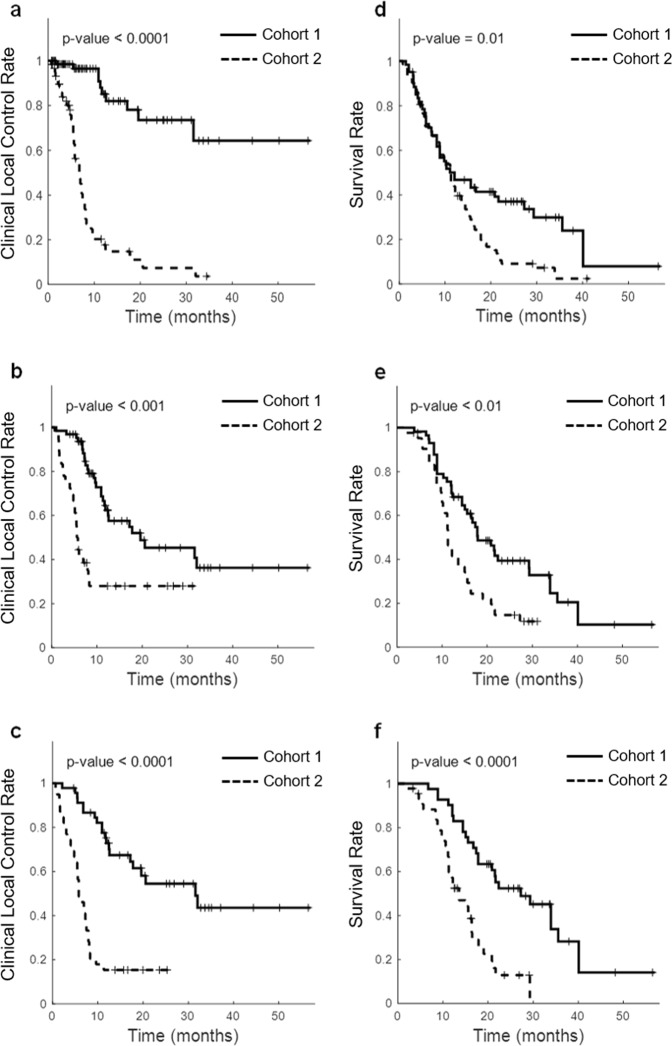
The Kaplan-Meier clinical local control and survival curves. The tumours were categorized into two cohorts based on the overall (**a**), 6-month (**b**), and 12-month (**c**) LC/LF predicted outcome at the first follow up (Cohort 1: predicted as LC; Cohort 2: predicted as LF using the qMRI optimal biomarkers). A patient was categorized into the LF cohort when they have one tumour with an overall (**d**), 6-month (**e**), and 12-month (**f**) LF predicted outcome at the first follow up.

The performance of the qMRI optimal biomarkers presented in Table 1 was further evaluated through long-term (five years) risk assessments in terms of the local outcome and overall survival using the Kaplan-Meier (KM) analysis. The risk assessment was performed for two separate cohorts: those predicted with a LC (cohort 1) versus LF (cohort 2) outcome by the radiomic framework. Figure 4 demonstrates the estimated long-term LC outcome of the tumours and overall survival of the patients. The plots show the KM clinical local control/survival curves obtained for the tumours/patients categorized based on the cross-validated 6-month, 12-month and overall LC/LF predicted outcomes at the first follow up using the optimal qMRI biomarkers. A statistically extremely significant difference (p-value < 0.001) was observed between the clinical local control curves of the two tumour cohorts categorized based on the optimal qMRI biomarkers of all three outcomes. Also, the survival curves obtained for the two patient cohorts categorized using the optimal qMRI biomarkers of the overall, 6-month, and 12-month outcomes demonstrated a statistically significant (p-value = 0.01), statistically highly significant (p-value < 0.01), and statistically extremely significant (p-value ≤ 0.001) difference, respectively.

## Discussion

In this study, the qMRI biomarkers derived from conventional T1w and T2-FLAIR images were investigated through a radiomics-machine learning framework to predict the local outcome in patients with brain metastasis treated with SRT. A total of 3072 geometrical and textural features were extracted from MR images of 100 patients (133 lesions) who underwent SRT for brain metastases. The features were extracted within the tumour, edema, tumour-margin, and lesion-margin before and at the first follow up after the treatment. The relative change from the baseline was calculated for each feature. The number of features was reduced to 927 using a correlation-based analysis. Subsequently, the best features were selected using Mann-Whitney U test in conjunction with a 50-fold sampling scheme, and a forward selection strategy to construct optimal biomarkers of overall, 6-month and 12-month LC/LF outcomes. The optimal qMRI biomarker consisted of 5 features for the overall LC/LF, and 4 features for the 6-month and 12-month local outcomes. All the selected features demonstrated a statistically significant, highly significant, or extremely significant difference between the lesions that had an outcome of LC versus LF.

The optimal biomarkers mainly consisted of features that characterize the heterogeneity within the edema, tumour-margin, and lesion-margin. Specifically, 11 out of the 13 distinct features in the three optimal biomarkers quantify changes in the heterogeneity in the region surrounding the tumour. These marginal regions are likely to contain malignant cells, but the number of these cells is not enough to result in an evident image contrast on standard MRI. The heterogeneity in these regions may characterize the frequency and distribution of cancerous cells and, therefore, could be linked to the LC/LF outcome of the treatment. This is in agreement with the findings of other studies in which the heterogeneity in the micro-structure of the tumour margin showed a good correlation to tumour response to therapy^54,55^.

Previous studies have demonstrated the prognostic power of the radiomics features extracted from ultrasound^56–60^, CT^61,62^, MR^63,64^, and pathology^65^ images. Such features have been reported to be predictive of different therapy outcomes (e.g. pathological response^66^, distant metastasis^51,61^, LC^50^, and OS^45^), and for various cancer sites including the lung^45,61^ brain^67^, breast^48,68^, and head and neck^51^ cancer. Whereas most previous studies have focused on the tumour itself, the results obtained from this study encourage conducting a comprehensive assessment of a larger region encompassing the entire lesion as well as its margin. Specifically, the qMRI biomarkers extracted from the edema and tumour/lesion margins demonstrated more prognostic power compared to features extracted from the tumour itself.

The results of the feature selection demonstrated a good potential of multi-wavelet filtered images for characterizing outcome-related attributes of metastatic lesions. In particular, four out of five, two out of four, and three out of four features selected in the optimal biomarkers of the overall, 6-month and 12-month LC/LF outcomes, respectively, were derived from the multi-wavelet filtered T1w or T2-FLAIR images. The multi-wavelet filtered images are obtained by filtering the original image in x, y, and z directions using the wavelet transform (Supplementary Figure 5). Such images contain the decomposed textural information of the original image in different bandwidths of spatial frequency for all directions. The results obtained in this study are in agreement with the findings of previous work that demonstrate the diagnostic and prognostic value of texture features extracted from wavelet images for tumour characterization and therapy outcome evaluation^45,61,65^. In this study, the features extracted from T2-FLAIR images demonstrated high prognostic power for prediction of LC/LF, where the top selected feature in all three optimal qMRI biomarkers (nine out of 13 selected features) were extracted from T2-FLAIR images. This observation further highlights the links between the characteristics of the edema micro-structure and LC in brain metastasis as T2-FLAIR images provide a better contrast for the edema region, compared to T1w images. Tumour size, SRT dose and number of fractions may impact LC/LF in brain metastasis. The average size of the tumours investigated in this study was 2.1 ± 1.1 cm, with a relatively small standard deviation. The majority of the tumours received a total dose of 30 Gy in 5 fractions. The total dose was adjusted to 25, 27.5, 32.5 or 35 Gy (in 5 fractions) for a few tumours based on their size. The LC/LF for each tumour was determined independent of the size, total dose and the number of fractions.

The optimal qMRI biomarkers were also applied to predict LF using an SVM classifier with the bootstrap 0.632 + and LOPO cross-validation methods. The optimal biomarkers could predict the overall, 6-month, and 12-month local outcomes with an ${\widehat{AUC}}_{.632+}$ of 0.79, 0.80, and 0.80 respectively. The cross-validated accuracy of the predictive model was found to be 80% for overall and 6-month LC/LF, and 82% for the 12-month LC/LF. The performance of the qMRI optimal biomarkers was further evaluated through long-term risk assessments using the KM analysis. A statistically significant difference was observed between the clinical local control curves, and survival curves obtained for the patients with a predicted LC versus LF outcome using the optimal qMRI biomarkers. The proposed model can potentially be used for clinical risk assessment, and treatment planning of brain metastasis patients. The results of the outcome prediction and risk assessment obtained in this study for 100 patients are promising. However, investigations on larger cohorts of patients (including an independent validation cohort) are required to assess further the efficacy and robustness of the technique in the clinic.

In conclusion, this study demonstrated a good potential of qMRI biomarkers derived from conventional T1w and T2-FLAIR images to predict the local outcome in metastatic brain tumours early after the treatment with SRT. The qMRI features identified in this study were (extremely/highly) significantly different between the tumours with a LF versus LC outcome. The proposed biomarkers were also found to have a good cross-validated sensitivity and specificity to distinguish the tumours with an outcome of LF within three months following the treatment. The promising results presented in this study imply that the qMRI biomarkers, as potential early surrogates of LC/LF in brain metastasis patients, can possibly facilitate effective changes in treatment on an individual patient basis that may improve patient outcomes. Patients predicted to have LF can potentially be treated with salvage surgical or radiation treatments earlier to prevent downstream morbidity of progressive metastatic disease in the brain.

## Methods

### Study protocol and data acquisition

This study was conducted in accordance with institutional research ethics approval from Sunnybrook Health Sciences Centre (SHSC), Toronto Canada (REB PIN: 294–2013). Imaging and clinical data were collected from 100 patients diagnosed with metastatic brain tumours and treated with SRT. The study only used retrospective data and the research ethics board granted a permission to do it without informed consent. Out of the 100 patients, 20 patients had 2, and 7 patients had 3 tumours, respectively. As such, the dataset included a total of 133 malignant lesions. The patients were scanned on a Philips 1.5 T Ingenia system (Best, Netherlands). Gadolinium-contrast-enhanced-T1-weighted (T1w) and T2-FLAIR images were acquired before (baseline) and at every follow up on a 2–3 month schedule after the SRT (for up to five years) as part of the institutional standard of care for these patients. The lesions were monitored longitudinally and the LC/LF outcome for each lesion was determined by a radiation oncologist and neuroradiologist using the follow up imaging data. The RANO-BM criteria was used to determine an outcome of LC (complete response, partial response, or stable disease) or LF (progressive disease) for each lesion^69^. Local progression was differentiated from radiation necrosis or adverse radiation effect (ARE) based on the report by Sneed *et al*.^70^. All cases of radiation necrosis were diagnosed based on serial imaging (including the use of perfusion MRI), and/or histological confirmation^71^. The overall LC/LF was defined as the local outcome identified in the last patient follow up within five years after the SRT. The 6-month and 12-month LC/LF were defined as the local outcome identified in the last follow up before 6 and 12 months after the radiation treatment, respectively. For the 6-month and 12-month LC/LF outcome analysis, the patients with a LC outcome who deceased before the end of follow-up window were excluded from the analysis. The median follow up and median OS for all patients were 8.3 and 11.3 months, respectively. Patient characteristics are summarised in Table 3.

**Table 3:** Summary of the patient characteristics.

Characteristic	Mean /Number [Range]/(Percentage)
Age	63 [21–92] years
Sex	Female: 63 (63%)
	Male: 37 (37%)
Initial Maximum Diameter of Tumour	2.1 [0.4–7] cm
Histology	Non-Small-Cell Lung Cancer: 65 (48.9%)
	Breast Cancer: 31 (23.3%)
	Melanoma: 12 (9.0%)
	Renal Cell Cancer: 9 (6.8%)
	Colorectal Cancer: 8 (6.0%)
	Other: 8 (6.0%)
Local Outcome	Overall LC: 80 (60.2%) | Overall LF: 53 (39.8%)
	6-month LC: 76 (76%) | 6-month LF: 24 (24%)
	12-month LC: 40 (47%) | 12-month LF: 45 (53%)

The radiomics framework in this study applied the images acquired at the baseline and the first follow up. The baseline images for each patient were acquired right before the radiation treatment planning, whereas the first follow-up images were acquired within three months after the treatment. The in-plane image resolution was 0.5 mm for both T1w and T2-FLAIR images. The slice thickness was 1.5 mm and 5 mm for T1w and T2-FLAIR images, respectively.

### Pre-processing and generation of sub-lesion masks

A linear interpolation was applied in the axial direction of T1w and T2-FLAIR images to make the voxel size isotropic (0.5 × 0.5 × 0.5 mm^3^). The interpolated images were used for image registration and in the generation of sub-lesion masks (described below). Each lesion was divided into four sub-regions: (1) the enhancing disease region in T1w images referred to hereafter as tumour, (2) edema, (3) a volume generated by an isotropic expansion around the tumour and edema referred to hereafter as lesion-margin, (4) a volume generated by an isotropic expansion around just the tumour referred to hereafter as tumour-margin (Fig. 1a). A treatment planning gross tumour volume (GTV) was contoured by expert radiation oncologists on baseline T1w images and used as a guide to generate GTVs on follow-up T1w images. Follow-up imaging GTVs were reviewed by an expert radiation oncologist (H.S.). A semi-automatic framework was developed to generate the sub-lesion binary masks for the T1w and T2-FLAIR images (Supplementary Figure 3). The T2-FLAIR images were initially registered to the corresponding T1w images using an affine registration method with mutual information (MI) as the similarity metric^72^. The tumour masks for T1w images were generated using the GTV contours at the baseline and the first follow up. To generate the edema masks for the T1w images, the edema region was segmented on the registered T2-FLAIR image using the region growing segmentation module in 3D slicer^73^. The automatic segmentations were further refined manually under supervision of the expert radiation oncologist. The inverse of the registration transformation matrix was used to warp the edema and tumour masks generated for the T1w images on the original T2-FLAIR images. Finally, the lesion-margin and tumour-margin masks were created using morphological image analysis. Four different margin sizes, including 1, 3, 5, and 10 mm, were investigated. A margin size of 3 mm for both the tumour- and lesion-margin was found to be associated with the best results that were reported here. Further details on the pre-processing and mask generation have been provided in the supplementary methods.

### Feature extraction

The quantitative features investigated in this study included two groups of geometrical and textural features. The geometrical features were calculated from the binary masks. The texture features were extracted from the T1w and T2-FLAIR images within the four sub-lesion masks. A total of 3072 geometrical and textural features were derived as described in detail in the supplementary information. The textural features included the first order histogram features derived from the MR images and the corresponding local binary pattern (LBP) parametric images, as well as the second order grey-level co-occurrence matrix (GLCM) features^74,75^. These features were extracted from both the original images and the multi-wavelet filtered images as described in the supplementary information^76^. The interpolated MR images with an isotropic voxel size were used to extract 3D features (GLCM and multi-wavelet features). The other 2D features were derived from the original images. All features were extracted from both the baseline and first follow up images and relative changes from the baseline at the first follow up were calculated.

### Feature reduction/selection

The optimal qMRI biomarkers for LC/LF outcome prediction were constructed using a multi-step feature reduction/selection procedure. The features were first assessed using a Pearson correlation analysis to obtain the coefficient of determination (R^2^) for each feature pair (Fig. 2a). Clusters of highly correlated features were identified from the R-squared matrix using a threshold of R^2^ = 0.8. Next, in each cluster of correlated features, the one with the largest dynamic range was selected as the representative feature. Using this approach, the 3072 features were reduced to 927. Subsequently, a two-step feature selection procedure was utilized to construct the optimal qMRI biomarkers. First, the features were ranked using the p-values obtained from the Mann-Whitney U test in conjunction with a 50-fold sampling scheme. In each sampling step, the p-values were calculated over 49 folds of the patients and the 15 features with the smallest p-values were identified. The features were then ranked using their frequency of occurrence in the best 15 features over the 50 samples. In the second step of feature selection, the ${\widehat{AUC}}_{.632+}$ was used with a forward feature selection scheme to find the feature sets leading to the maximum performance. The ${\widehat{AUC}}_{.632+}$ is the average of the $AU{C}_{.632+}$ values obtained for all bootstrap 0.632 + samples drawn from the data set. The $AU{C}_{.632+}$ is a weighted sum of the re-substitution and test AUCs computed for each bootstrap sample (supplementary information). In the forward feature selection, the ${\widehat{AUC}}_{.632+}$ was calculated for each feature set using 250 bootstrap samples and an SVM classifier. The best feature set obtained from the forward selection was further reduced using an ANOVA test to find the smallest set of features (referred to as the optimal qMRI biomarker) leading to a statistically similar performance. The details of feature reduction/selection are described in supplementary information. The proposed feature selection procedure was performed independently for each of the overall, 6-month, and 12-month LC/LF outcomes.

### Outcome prediction and risk assessment

The outcomes prediction was performed independently after the feature selection step using the optimal qMRI biomarkers. An SVM classifier was used in conjunction with bootstrap 0.632 + and LOPO cross-validation methods to predict the LC/LF outcomes. The ${\widehat{AUC}}_{.632+}$ was calculated using another 250 bootstrap samples. A Gaussian kernel was used in the SVM for predicting the overall and 12-month outcomes. A linear SVM kernel was used for the 6-month outcome prediction. In order to compensate for the imbalance of the dataset, a random under-sampling was applied on the majority class (LC outcome) before each bootstrap sampling^77^. The under-sampling was applied on a patient level to have a balanced sample with an equal number of lesions with LC and LF outcomes.

The accuracy, sensitivity, and specificity of the model were calculated using a LOPO cross-validation. Each LOPO cross-validation step involved leaving one patient out, training the SVM classifier independently on 250 bootstrap samples from the training set, and characterizing the test lesions (associated with the left-out patient) with a majority vote of the 250 trained classifiers. The classifier’s parameters were similar to those used for calculating the ${\widehat{AUC}}_{.632+}$.

Long-term risk analyses were performed to generate Kaplan-Meier clinical local control and survival curves for the two cohorts with different predicted outcomes. The overall, 6-month, and 12-month predicted outcomes (LC/LF) for each lesion was determined using the LOPO cross validation scheme. A patient was categorized into the LF cohort in case they have one tumour with a cross-validated LF predicted outcome at the first follow up. The Kaplan-Meier curves were compared using a log-rank test to assess for statistically significant differences between the clinical local control (lesion level) and survival (patient level) curves.

## Supplementary information

Supplementary Information

## Data Availability

Data were collected and available at the Odette Cancer Centre, Sunnybrook Health Sciences Centre, Toronto, ON, Canada.
