# Supplementary material for: Quantitative MRI Biomarkers of Stereotactic Radiotherapy Outcome in Brain Metastasis
Source: Sci Rep. 2019 Dec 27;9:19830. doi: 10.1038/s41598-019-56185-5 (PMC6934477; doi:10.1038/s41598-019-56185-5)
Supplement: Supplementary file 1 — Supplementary Information [file 41598_2019_56185_MOESM1_ESM.pdf]

Supplementary Information

Quantitative MRI Biomarkers of Stereotactic Radiotherapy Outcome in Brain Metastasis

Elham Karami[1,2,3], Hany Soliman[4,5], Mark Ruschin[4,5], Arjun Sahgal[4,5], Sten Myrehaug[4,5], Chia-Lin Tseng[4,5], Gregory J. Czarnota[2,3,4,5], Pejman Jabehdar-Maralani[6], Brige Chugh[4,5], Angus Lau[2,3,4], Greg J. Stanisz[2,3,7], and Ali Sadeghi-Naini[1,2,3,4]*

(1) Department of Electrical Engineering and Computer Science, Lassonde School of Engineering, York University, Toronto, ON, Canada
(2) Department of Medical Biophysics, University of Toronto, Toronto, ON, Canada
(3) Physical Sciences Platform, Sunnybrook Research Institute, Sunnybrook Health Sciences Centre, Toronto, ON, Canada
(4) Department of Radiation Oncology, Odette Cancer Centre, Sunnybrook Health Sciences Centre, Toronto, ON, Canada
(5) Department of Radiation Oncology, University of Toronto, Toronto, ON, Canada
(6) Department of Medical Imaging, Sunnybrook Health Sciences Centre, Toronto, ON, Canada
(7) Department of Neurosurgery and Pediatric Neurosurgery, Medical University, Lublin, Poland

* Corresponding author:
Dr. Ali Sadeghi-Naini
Department of Electrical Engineering and Computer Science
Lassonde School of Engineering, York University
LAS 3047, Lassonde Building
4700 Keele Street, Toronto, ON, Canada, M3J 1P3
Email: asn@yorku.ca

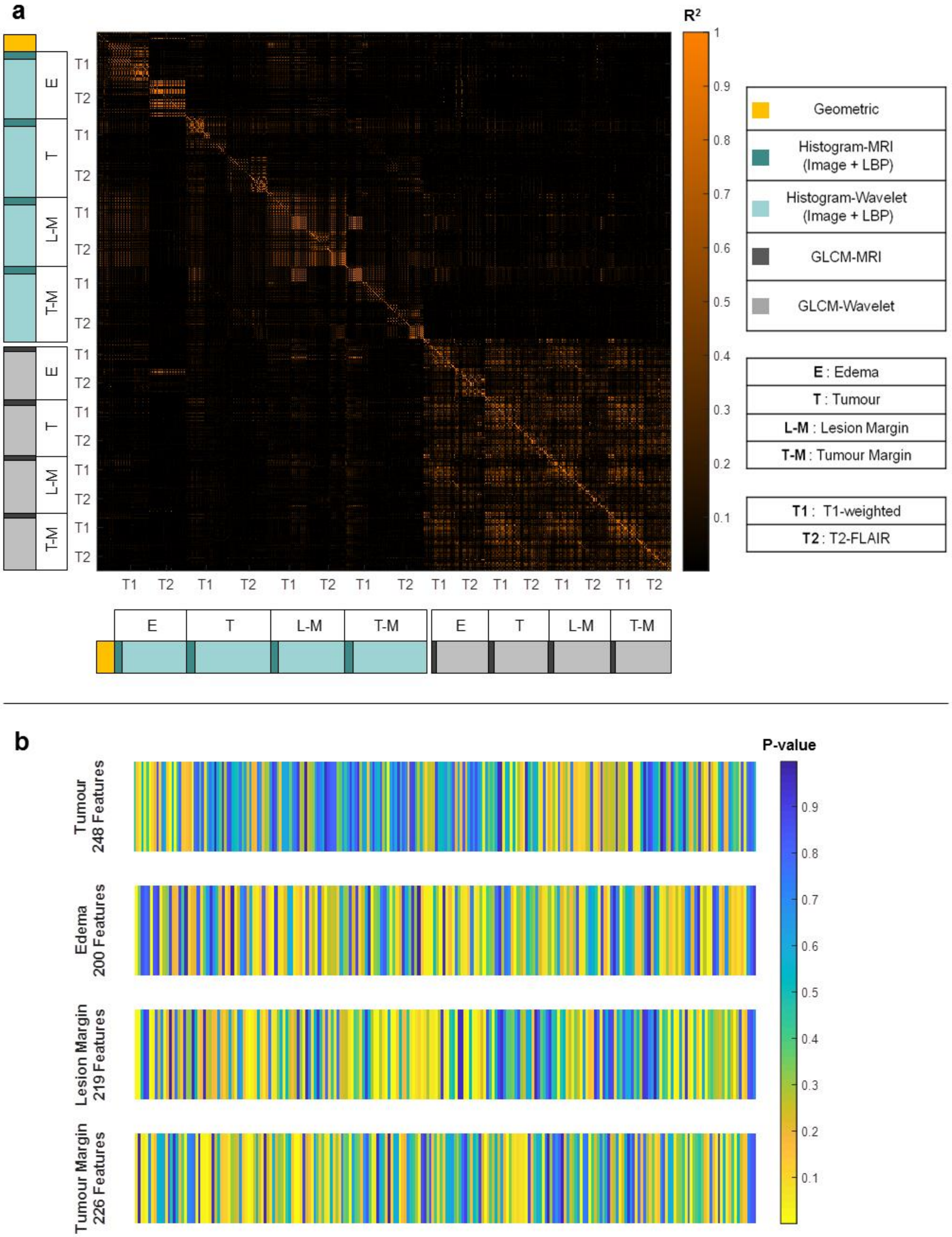

Figure 1 | The qMR feature heat maps for 6-month LC/LF. (a) The R-squared heat map generated using the Pearson correlation coefficients for all pairs of the extracted features. **(b)** The p-value heat maps for different sub-regions of the lesion generated using the Mann-Whitney U test (6 month LC/LF) for each feature after the redundant features were eliminated.

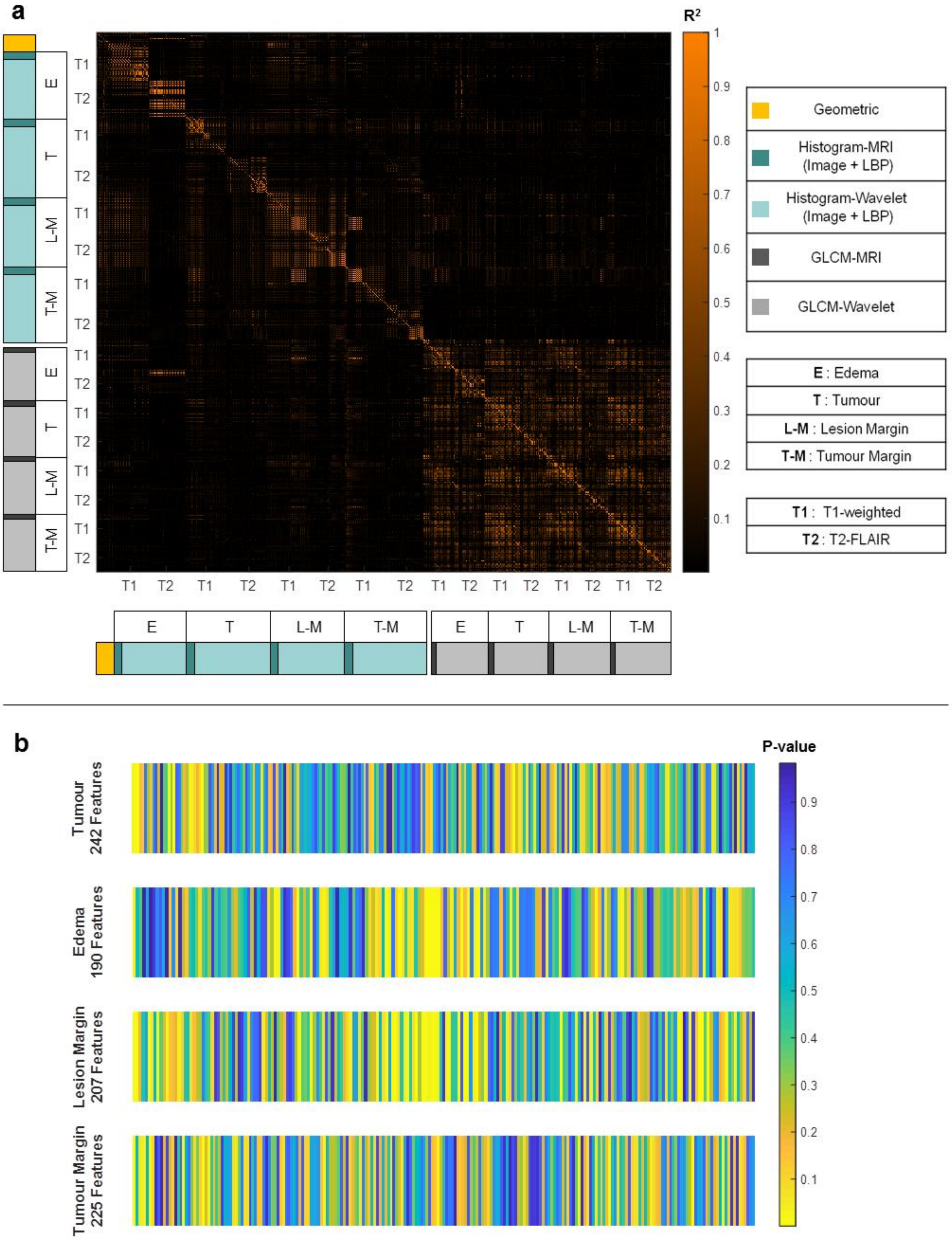

Figure 2 | The qMR feature heat maps for 12-month LC/LF. (a) The R-squared heat map generated using the Pearson correlation coefficients for all pairs of the extracted features. **(b)** The p-value heat maps for different sub-regions of the lesion generated using the Mann-Whitney U test (12 month LC/LF) for each feature after the redundant features were eliminated.

Image Registration/Segmentation and Mask Generation

To obtain the regions of interest (ROIs) for sub-lesion mask generation, a semi-automatic registration-segmentation framework was developed (Figure 1). The baseline T2-weighted-fluid-attenuation-inversion-recovery (T2-FLAIR) images were initially registered to their corresponding Gadolinium-contrast-enhanced-T1-weighted (T1w) images using an affine registration method with mutual information as the metric[1]. The edema region was segmented on the registered T2-FLAIR images using a region growing algorithm[2]. The region growing segmentation was followed by manual refinements performed in 3D slicer[3]. Finally, the inverse of the registration transformation matrix was used to warp the edema, and the tumour masks generated for the T1w images on the T2-FLAIR images. A similar procedure was performed on the follow-up images for generation of the sub-lesion masks. In addition to the tumour and edema masks, the lesion-margin and tumour-margin masks were generated using morphological image analysis, covering a 1, 3, 5, and 10 mm margin of the lesion (tumour + edema), and tumour, respectively.

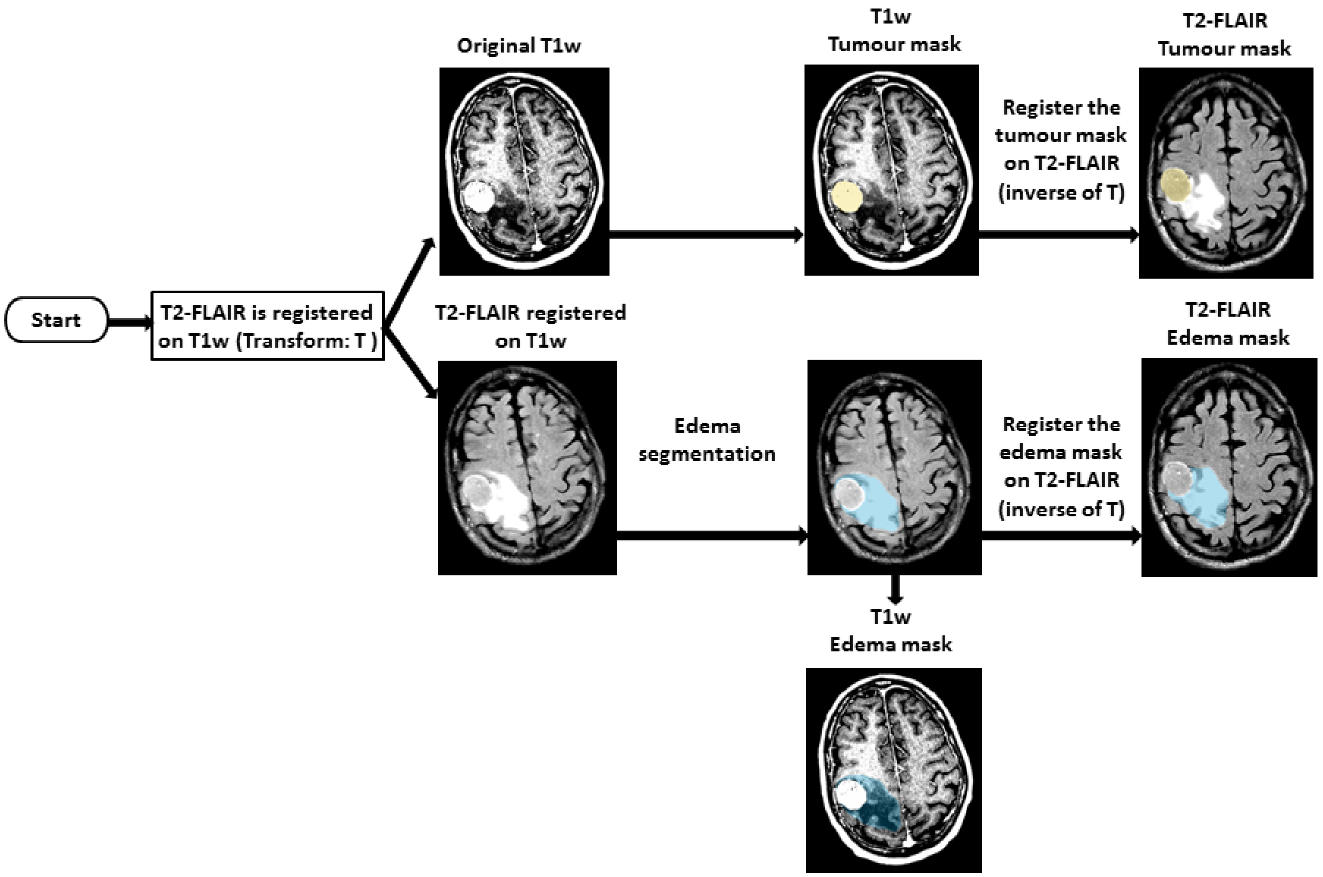

Figure 3 | The mask generation framework for the T1w and T2-FLAIR images.

Radiomics Features

A total number of 3072 were extracted from the segmented ROIs. The derived features included four groups of: 1) geometrical features, 2) histogram features, 3) gray-level co-occurrence matric (GLCM) features, and 4) multi-wavelet features.

1) Geometrical Features

The geometrical features were extracted from 2D axial slices of the binary masks. The extracted features were subsequently averaged over all axial slices. The following geometrical features were extracted using the MATLAB software package:

1. Area: The area of the ROI calculated using the number of pixels multiplied by the area of each pixel (S)
2. Perimeter: The perimeter of the ROI (P)
3. Circle Metric

$$\text{circle metric} = \frac{P^2}{4\pi S}$$

4. Aspect Ratio

$$\text{aspect ratio} = \frac{\text{width of ROI}}{\text{height of ROI}}$$

5. Convexity

$$\text{convexity} = \frac{\text{The perimeter of convex hull}}{P}$$

6. Eccentricity: Eccentricity of the ellipse that has the same second-moments as the ROI. The eccentricity is the ratio of the distance between the foci of the ellipse and its major axis length.

7. Solidity

$$\text{solidity} = \frac{S}{\text{convex area}}$$

8. Extent

$$\text{extent} = \frac{S}{\text{area of the bounding box}}$$

2) Histogram Features

The histogram statistics describe the distribution of voxel intensities within an image. In this study, the histogram features were extracted from both the MR images and the corresponding local binary pattern (LBP) parametric images (described below).

The histogram features were computed for each 2D image slice, and subsequently averaged over all 2D slices. The following first-order statistical features were extracted:

1. Mean

$$\mathrm{mean} = \frac{1}{N}\sum_{i=1}^{N} X(i)$$

2. Variance

$$\mathrm{variance} = \frac{1}{N-1}\sum_{i=1}^{N} (X(i) - \overline{X})^2$$

3. Skewness

$$\mathrm{skewness} = \frac{\frac{1}{N}\sum_{i=1}^{N}(X(i) - \overline{X})^3}{(\sqrt{\frac{1}{N}\sum_{i=1}^{N}(X(i) - \overline{X})^2})^3}$$

4. Kurtosis

$$\mathrm{kurtosis} = \frac{\frac{1}{N}\sum_{i=1}^{N}(X(i) - \overline{X})^4}{(\sqrt{\frac{1}{N}\sum_{i=1}^{N}(X(i) - \overline{X})^2})^2}$$

5. Energy

$$\mathrm{energy} = \sum_{i=1}^{N} X(i)^2$$

6. Entropy

$$\mathrm{entropy} = \sum_{i=1}^{N_l} \mathbf{P}(i) \log_2 \mathbf{P}(i)$$

7. Maximum

$$\mathrm{maximum} = \max_{i=1,\dots,N} X(i)$$

8. Mean Absolute Deviation

$$\text{mean absolute deviation} = \frac{1}{N}\sum_{i=1}^{N} \|X(i) - \bar{X}\|$$

9. Median: The median intensity value.

10. Minimum

$$\text{minimum} = \min_{i=1,\ldots,N} X(i)$$

11. Range

$$\text{range} = \text{maximum} - \text{minimum}$$

12. Root Mean Square (RMS)

$$\text{RMS} = \sqrt{\frac{\sum_{i}^{N} X(i)^2}{N}}$$

13. Standard Deviation (SD)

$$\text{SD} = (\frac{1}{N-1}\sum_{i=1}^{N}((X(i) - \bar{X})^2)^{1/2}$$

14. Uniformity

$$\text{uniformity} = \sum_{i=1}^{N_l} P(i)^2$$

X(i) represents the intensity value of pixel i, and P(i) is the probability of the intensity value i.

LBP

LBP is a very efficient texture operator which labels the pixels of an image by thresholding the neighborhood of each pixel and considers the result as a binary number[4]. The LBP feature vector, in its simplest form, is created in the following manner:

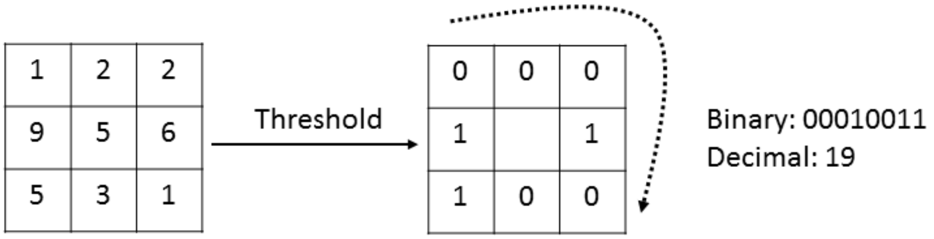

Figure 4 | Thresholding the pixel neighborhood to obtain the LBP feature.

To obtain the LBP feature for each pixel, the examined window is divided into cells (*e.g.* 16×16 pixels for each cell). Next, for each pixel in a cell the pixel value is compared to each of its 8 neighbors (on its left-top, left-middle, left-bottom, right-top, etc.). The comparison is performed along a circular path, *i.e.* clockwise or counter-clockwise. In general, different neighbourhoods can be defined by varying the radius of the circle around the pixel (R) and the quantisation of the angular space (Q). In case the center pixel's value is greater than the neighbor's value, the neighbor's value is changed to "0". Otherwise, it is changed to "1". For a neighbourhood consisting of 8 pixels this procedure gives an 8-bit number, which is usually converted to decimal for convenience. Next, a histogram representing the frequency of occurrence of each (8-bit) number is computed over the cell. For a neighbourhood consisting of 8 pixels, this histogram can be seen as a 256-dimensional feature vector that is often normalized to the number of pixels in the cell. Finally, the histogram of all cells are concatenated which gives a feature vector (parametric image) for the entire window.

3) Gray-Level Co-occurrence Matrix (GLCM) Features

The GLCM features are second-order texture features describing the relative position of the various gray-level intensities over the image that are computed using the gray level co-occurrence matrix[5]. To compute the gray level co-occurrence matrix, voxel intensities were linearly quantized using a bin-width of 25 gray levels [6]. The discretization step helps with noise removal and consistent texture extraction among all patients.

The gray-level co-occurrence matrix is a $N_g \times N_g$ matrix defined as $P(i, j, \delta, \alpha)$. The entry (i, j) of P is computed by finding the relative number of times (probability) that j occurs in direction α, and distance of δ pixels from i. Here, N_g is the number of discrete gray level intensities.

In this study, δ was a distance of 1 to 4 pixels, and α was each of the 13 directions in a 3D matrix (45° rotation between the adjacent directions). The final GLCM matrix was the accumulation of all the GLCM matrices calculated for each δ, and α.

For the co-occurrence matrix $P(i,j)$ with distance δ, direction α, and number of intensity levels N_g, the following parameters are calculated:

μ, the mean of $P(i,j)$,

$p_x(i) = \sum_{j=1}^{N_g} \mathbf{P}(i,j)$, and $p_y(i) = \sum_{i=1}^{N_g} \mathbf{P}(i,j)$ the marginal row, and column probabilities respectively,

μ_x, and σ_x the mean and standard deviation of p_x,

μ_y, and σ_y the mean and standard deviation of p_y,

$p_{x+y}(k) = \sum_{i=1}^{N_g} \sum_{\boldsymbol{j=1}}^{N_g} \mathbf{P}(i,j)\, , i + j = k, k = 2,3, \dots ,2N_g,$

$p_{x-y}(k) = \sum_{i=1}^{N_g} \sum_{\boldsymbol{j=1}}^{N_g} \mathbf{P}(i,j)\, , |i - j| = k, k = 2,3, \dots ,2N_g,$

$HX = -\sum_{i=1}^{N_g} p_x\,(i) \log_2[p_x(i)]$ be the entropy of p_x,

$HY = -\sum_{i=1}^{N_g} p_y\,(i) \log_2[p_y(i)]$ be the entropy of p_y,

$H = -\sum_{i=1}^{N_g} \sum_{j=1}^{N_g} P(i,j) \log_2[P(i,j)]$ be the entropy of $P(i,j)$,

$HXY1 = -\sum_{i=1}^{N_g} \sum_{j=1}^{N_g} \mathbf{P}(i,j) \log(p_x(i) p_y(j)),$

$HXY2 = -\sum_{i=1}^{N_g} \sum_{j=1}^{N_g} p_x(i) p_y(j) \log(p_x(i) p_y(j)).$

The texture features are then computed using the following formula:

1. Autocorrelation:

$$\text{autocorrelation} = \sum_{i=1}^{N_g} \sum_{j=1}^{N_g} ij\mathbf{P}(i,j)$$

2. Cluster Prominence

$$\text{cluster prominence} = \sum_{i=1}^{N_g}\sum_{j=1}^{N_g}\left[i + j - \mu_x(i) - \mu_y(j)\right]^4 \mathbf{P}(i,j)$$

3. Cluster Shade

$$\text{cluster shade} = \sum_{i=1}^{N_g}\sum_{j=1}^{N_g}[i + j - \mu_x(i) - \mu_y(j)]^3 \mathbf{P}(i,j)$$

4. Contrast

$$\text{contrast} = \sum_{i=1}^{N_g}\sum_{j=1}^{N_g}[i - j]^2 \mathbf{P}(i,j)$$

5. Correlation

$$\text{correlation} = \frac{\sum_{i=1}^{N_g}\sum_{j=1}^{N_g} ijP(i,j) - \mu_i(i)\mu_j(j)}{\sigma_x(i)\sigma_y(j)}$$

6. Difference Entropy

$$\text{difference entropy} = \sum_{i=0}^{N_g-1} P_{x-y}(i)\log_2[P_{x-y}(i)]$$

7. Dissimilarity

$$\text{dissimilarity} = \sum_{i=1}^{N_g}\sum_{j=1}^{N_g}|i - j|\,\mathbf{P}(i,j)$$

8. Energy

$$\text{energy} = \sum_{i=1}^{N_g}\sum_{j=1}^{N_g}[\mathbf{P}(i,j)]^2$$

9. Entropy

$$\text{entropy} = -\sum_{i=1}^{N_g}\sum_{j=1}^{N_g}\mathbf{P}(i,j)\log_2[\mathbf{P}(i,j)]$$

10. Homogeneity

$$\text{homogeneity} = \sum_{i=1}^{N_g}\sum_{j=1}^{N_g}\frac{\mathbf{P}(i,j)}{1 + |i - j|}$$

11. Informational Measure of Correlation 1 (IMC1):

$$\mathrm{IMC1} = \frac{\mathrm{HXY} - \mathrm{HXY1}}{\max(\mathrm{HX}, \mathrm{HY})}$$

12. Information Measure of Correlation 2 (IMC2):

$$\mathrm{IMC2} = \sqrt{1 - e^{-2(\mathrm{HXY2} - \mathrm{HXY})}}$$

13. Inverse Difference:

$$\text{inverse difference} = \sum_{i=1}^{N_g} \sum_{j=1}^{N_g} \frac{\mathbf{P}(i, j)}{1 + (\frac{|i - j|}{N})}$$

14. Maximum Probability

$$\text{maximum probability} = \max\{\mathbf{P}(i, j)\}$$

15. Sum Average

$$\text{sum average} = \sum_{i=2}^{2N_g} [i\mathbf{P}_{x+y}(i)]$$

16. Sum Entropy

$$\text{sum entropy} = -\sum_{i=2}^{2N_g} \mathbf{P}_{x+y}(i) \log_2[\mathbf{P}_{x+y}(i)]$$

17. Sum Variance

$$\text{sum variance} = \sum_{i=2}^{2N_g} (i - SE)^2 \mathbf{P}_{x+y}(i)$$

18. Variance

$$\text{variance} = \sum_{i=1}^{N_g}\sum_{j=1}^{N_g}(i-\mu)^2\,\mathbf{P}(i,j)$$

4) Wavelet Features

The last group of features were derived from multi-wavelet filtered images which were acquired by filtering the MR images in x, y, and z directions using the wavelet filters[7]. The wavelet filters extract textural information by decomposing the image in low (L), and high (H) frequencies. The procedure for applying the three dimensional wavelet transform on each image is shown in Figure 5 where the first layer corresponds to filtering along the x axis. The second and thirds layers correspond to filtering along the y, and z axes, respectively. Once the multi-wavelet images were computed, the histogram and GLCM features were extracted from each multi-wavelet image.

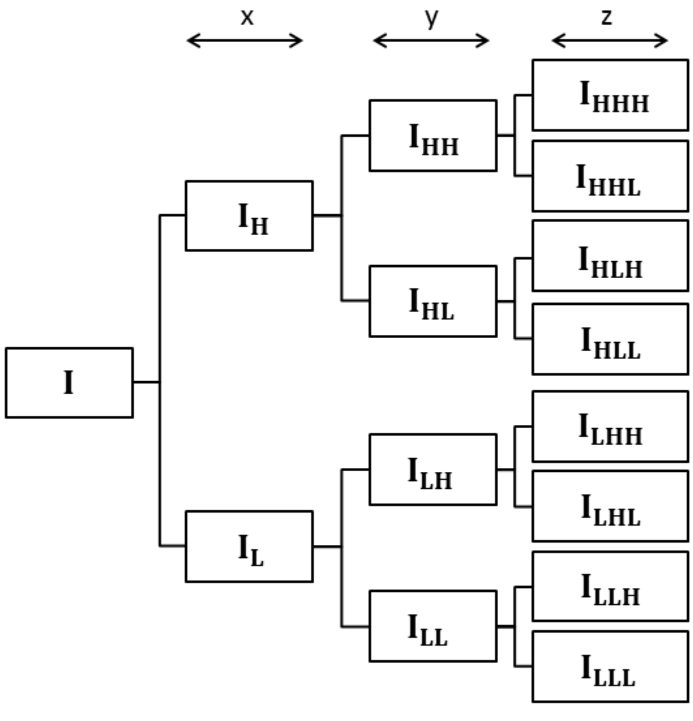

Figure 5 | Multi-wavelet filtering.

Feature Reduction/Selection

The optimal features for LC/LF prediction were obtained through a multi-step feature reduction/selection procedure. The features reduction procedure involved using a Pearson correlation analysis to obtain the coefficient of determination (R^2) for each feature pair (Figures 1 and 2). Clusters of highly correlated features were identified from the R-squred matrix using a threshold of $R^2 = 0.8$. Next, in each cluster of correlated features, the one which was associated with the largest dynamic range was selected as the representative feature. The feature reduction step was followed by a two-step feature selection procedure. First, the features were ranked using the p-values obtained from the Mann-Whitney U test in conjunction with a 50-fold sampling scheme. In each sampling step, the p-values were calculated over 49 folds of the patients and the 15 features with the smallest p-values were identified. The features were then ranked using their frequency of occurrence over the 50 samples. In the second step of feature selection, the $\widehat{AUC}_{.632+}$ was used with a forward feature selection scheme to find the feature sets leading to the maximum performance.

The $\widehat{AUC}_{.632+}$ is the average of the $AUC_{.632+}$ values obtained for all bootstrap .632+ samples drawn from the dataset X. In this study, a balancing step was performed before each bootstrap sampling to compensate for the imbalance of the dataset. For the dataset X consisting of N observations, let $\mathrm{X^{bal}}$ be a balanced set obtained by keeping all the observations from the minority group (n=N1), and random sampling of N1 observations from the majority group. Next, let $\mathrm{X_{train}^{bal-b}}$ be the selected bootstrap training sample at step b $(\mathrm{b} = 1, 2 \ldots \mathrm{B})$, and $\mathrm{X_{test}^{bal-b}}$ the corresponding test sample obtained by excluding the training samples from $\mathrm{X^{bal}}$.

For each step, the bootstrap AUC, $\mathrm{AUC_b}$, can be obtained by testing the trained classifier on the bootstrap test set. $\mathrm{AUC_b}$ is known to be upward biased. As such, the bias is accounted for using the following definition:

$$\mathrm{AUC_{.632+}^{b}} = \alpha_\mathrm{b}\mathrm{AUC_b'} + (1 - \alpha_\mathrm{b})\mathrm{AUC_x},$$

where

$$\mathrm{AUC_b'} = \max\{0.5, \mathrm{AUC_b}\},$$

$$\alpha_\mathrm{b} = \frac{0.632}{1 - 0.368 \times \mathrm{R_b}},$$

$$R_b = \begin{cases} 1 & \text{if } AUC_b \leq 0.5 \\ \dfrac{AUC_x - AUC_b}{AUC_x - 0.5} & \text{if } AUC_x > AUC_b > 0.5 \\ 0 & \text{otherwise,} \end{cases}$$

and AUC_x is the AUC obtained from testing the trained classifier on the trained set (re-substitution AUC). AUC_x is known to be downward biased. Finally, the overall AUC for bootstrapping test is defined as:

$$\widehat{AUC}_{.632+} = \frac{1}{B}\sum_{b=1}^{B} AUC_{.632+}^{b}.$$

In the forward feature selection, the $\widehat{AUC}_{.632+}$ was calculated for each feature set using 250 bootstrap samples and an SVM classifier. The best feature set obtained from the forward selection was further reduced using an ANOVA test to find the smallest set of features (referred to as the optimal qMR biomarker) leading to a statistically similar performance. Specifically, in each step of forward selection 250 values were computed for $AUC_{.632+}^{b}$, and M features resulting in maximum $\widehat{AUC}_{.632+}$ were selected. The ANOVA test was performed on the M vectors of $AUC_{.632+}^{b}$ values, corresponding to the M selected features, to find the feature sets for which the $AUC_{.632+}^{b}$ values are significantly different. The feature selection procedure is demonstrated in Figure 4. The feature extraction, reduction, selection and classification algorithms were implemented in MATLAB software package.

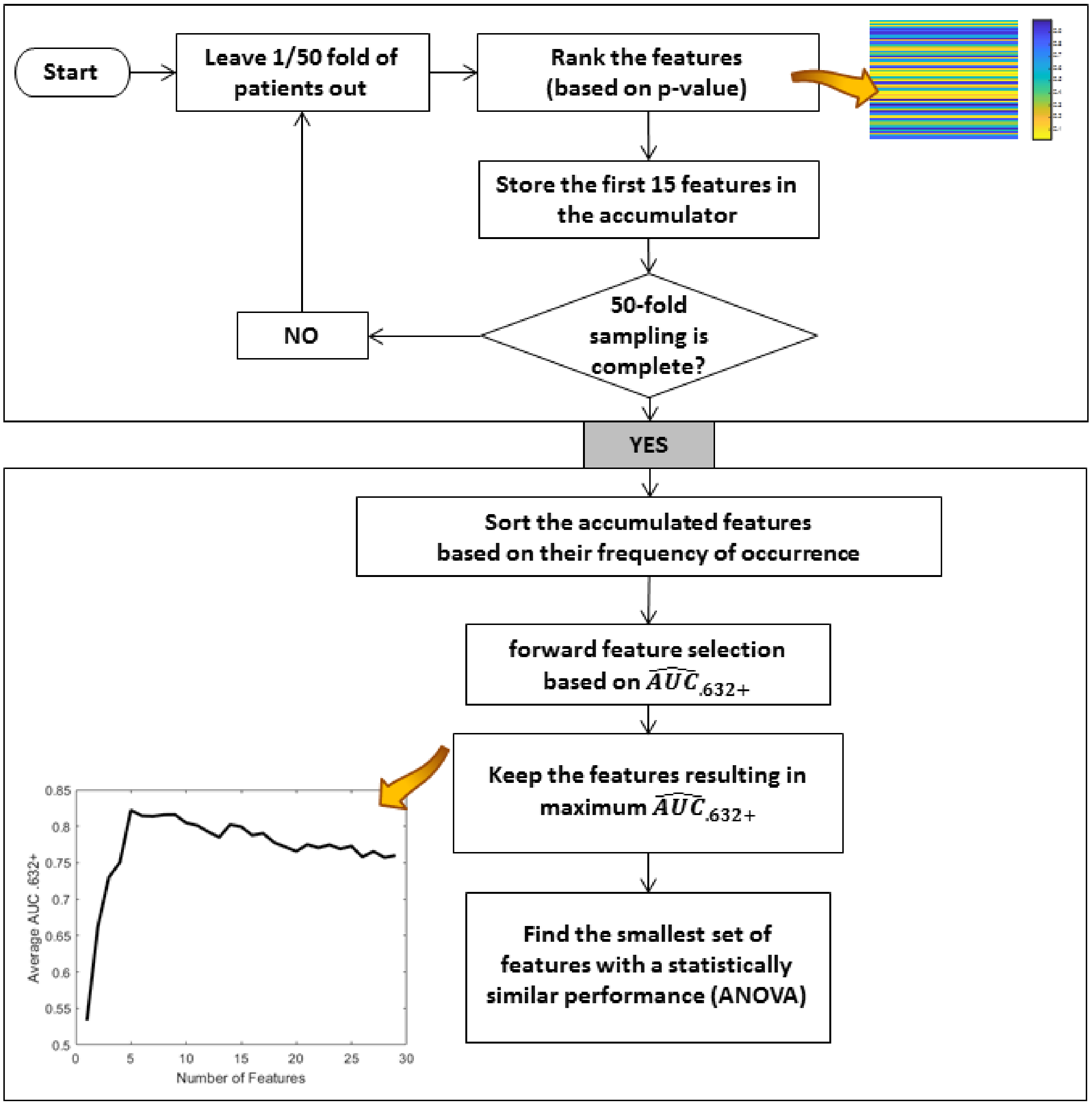

Figure 6 | The feature selection algorithm.

References

1. Hill, D. L. G., Batchelor, P. G., Holden, M. & Hawkes, D. J. Medical image registration. *Physics in Medicine and Biology* **46**, 173–178 (2001).
2. Gonzalez, R. C. & Woods, R. E. *Digital Image Processing*. (Pearson, 2007).
3. Fedorov, A. *et al.* 3D Slicer as an image computing platform for the Quantitative Imaging Network. *Magn. Reson. Imaging* **30**, 1323–1341 (2012).
4. He, D. C. & Wang, L. Texture Unit, Texture Spectrum, and Texture Analysis. *IEEE Trans. Geosci. Remote Sens.* **28**, 509–512 (1990).
5. Haralick, R. M., Shanmugam, K. & Dinstein, I. Textural Features for Image Classification. *IEEE Trans. Syst. Man. Cybern.* **3**, 610–621 (1973).
6. Aerts, H. J. W. L. *et al.* Decoding tumour phenotype by noninvasive imaging using a quantitative radiomics approach. *Nat. Commun.* **5**, 4006 (2014).
7. Graps, A. An introduction to wavelets. *IEEE Comput. Sci. Eng.* **2**, 50–61 (1995).
